# Renewal of challenging behavior in an intensive outpatient clinic: Replication and extension to task changes

**DOI:** 10.1002/jaba.70057

**Published:** 2026-02-11

**Authors:** Ishita Aggarwal, Matthew J. O'Brien, Alexander M. Pauls, Sara R. Jeglum, Christopher T. Franck, Carla N. Martinez‐Perez, Christopher A. Podlesnik

**Affiliations:** ^1^ Stead Family Department of Pediatrics University of Iowa Iowa City IA USA; ^2^ Department of Psychological and Quantitative Foundations University of Iowa Iowa City IA USA; ^3^ Blank Children's Hospital Des Moines IA USA; ^4^ Department of Statistics Virginia Tech University Blacksburg VA USA; ^5^ Department of Psychology University of Florida Gainesville FL USA

**Keywords:** behavior therapy, challenging behavior, consecutive case series, lapse, renewal

## Abstract

Retrospective analyses have shown that renewal of challenging behavior following person or setting changes is common. This study replicates and extends prior work by evaluating renewal after a third type of context change: task changes. Among 98 patients in an intensive outpatient clinic who experienced 749 context changes, overall renewal prevalence was 25.23% using a max‐of‐5 criterion (39.79% using a mean‐of‐2 criterion), consistent with prior reports across this large sample. Of the 63 patients who experienced at least one task change, 36 (57.14%) exhibited renewal. Task changes produced higher renewal rates than person or setting changes across criteria, but differences were not statistically significant. Renewal magnitude generally declined across sessions; however, challenging behavior rarely returned to prechange levels, even after five sessions. Findings highlight the broader range of contextual variables that may evoke renewal and the need for strategies to reduce its intensity and persistence during treatment.

Renewal is a form of behavioral lapse^1^ characterized by a return of previously reduced behavior resulting from a change in context (Bouton et al., 2012; Brown, 2025; Podlesnik et al., 2017). Although most studies to date have involved nonhumans in basic research (Saini & Mitteer, 2020), renewal has recently drawn more attention by applied researchers. Several retrospective analyses of renewal within clinical settings have focused on the prevalence of renewal following changes across people (i.e., treatment implementer, such as therapist, caregiver, staff, teacher) and settings (e.g., clinic, home, school; Falligant et al., 2021, 2022, 2024; Mitteer et al., 2022; Muething et al., 2020, 2022). In general, these studies have revealed renewal to be a concern during the treatment of challenging behavior across individuals diagnosed with neurodevelopmental and behavioral disorders. Renewal is a critically important topic for clinicians and researchers who implement treatments for challenging behavior, as context changes are common and necessary during treatment. For example, staffing changes are routine within behavior clinics, resulting in programmed and unprogrammed changes in treatment implementers. Studies evaluating the prevalence of renewal are important for understanding the scope of the problem in clinical settings, and they also provide a baseline estimate from which to measure the effects of efforts to mitigate renewal.

Muething et al. (2020) conducted the first retrospective analysis of renewal of challenging behavior in a clinical setting. They evaluated challenging behavior for 67 individuals (ages 4–21 years) with developmental disabilities in an outpatient clinic. Muething et al. defined prevalence as the frequency of renewal events divided by the total number of context changes in the sample. They showed renewal was relatively common, occurring across 42.31% of all context changes.

Several subsequent retrospective studies have since been published with similar populations across a variety of clinical settings. Table 1 summarizes key characteristics and outcomes of these studies. Although the overall prevalence in these studies has been lower than that observed in Muething et al. (2020), ranging from 23.50% (Falligant et al., 2021) to 30.54% (Falligant et al., 2022), the findings suggest that renewal is relatively common in clinics treating challenging behavior and similarly problematic following person and setting changes.

**TABLE 1:** Summary of outcomes from prior renewal studies and the present study.

Study	Function of challenging behavior	Clinic type	N	Population (diagnosis and age)	Percentage of cases with renewal (no. of cases)	Overall prevalence (total no. of context changes)	Prevalence for person changes (no. of person changes)	Prevalence for setting changes (no. of setting changes)	Prevalence for task changes (no. of task changes)
Muething et al. (2020)	Social	DT	67	IDD; child and young adult	67.16% (45)	42.31% (182)	42.19% (64)	42.37% (118)	N/A
Falligant et al. (2021)	Social	IP	34	IDD; child and young adult	58.82% (20)	23.50% (200)	24.71% (170)	16.67% (30)	N/A
Falligant et al. (2022)	Social	IOP	47	IDD; child and adult	59.57% (28)	30.54% (167)	38.71% (31)	28.68% (136)	N/A
Mitteer et al. (2022)	Social	IOP + DT	10	Various NDDs or DBDs; child and young adult	88.89% (9)	30.19% (53)	NR	NR	N/A
Present study^a^	Social	IOP	98	Various NDDs or DBDs; child and adult	71.43% (70)	25.23% (749)	24.69% (405)	22.92% (96)	27.02% (248)

*Note*: DT = day treatment; IP = inpatient; IOP = intensive outpatient; IDD = intellectual and developmental disabilities; NDD = neurodevelopmental disorder; DBD = disruptive behavior disorder; NR = not reported; no. = number.

^a^ Data using max‐of‐5 criterion only.

To date, studies of renewal using large clinical data sets have considered person and setting changes as context changes. However, basic research has demonstrated that changes to a wide range of contextual stimuli may produce renewal effects (for a review, see Bouton, 2019). Relatedly, several applied studies have demonstrated that changes to the type of task used in a behavioral treatment may affect treatment effectiveness (Durand & Carr, 1991; Shore et al., 1994; Wacker et al., 2005). For example, Wacker et al. (2005) implemented functional communication training (FCT) with 25 young children with developmental disabilities and challenging behavior to examine generalization across people, settings, and tasks. FCT produced robust treatment effects. When generalization was assessed posttreatment, improvements reliably occurred across people (e.g., parents, therapists, teachers) and settings (e.g., classrooms, living rooms, kitchens). In contrast, generalization to novel tasks (e.g., brushing teeth, academic work, cleaning up toys) was notably weaker, suggesting that task changes may pose a greater challenge to treatment durability than changes in implementer or setting.

Numerous studies have demonstrated differential effects of task type on challenging behavior, often attributable to task‐specific effects such as changes in response effort or reinforcement rate (e.g., DePaepe et al., 1996; Hagan‐Burke et al., 2015; Lee et al., 1999; Zangrillo et al., 2020). However, no studies to date have systematically evaluated the prevalence or magnitude of renewal following task changes that occur when reinforcement contingencies are otherwise held constant. It therefore remains unclear whether task changes represent a threat to renewal that is similar to that of changes in the treatment implementer or setting. This represents an important gap given how often task variation occurs in treatments for escape maintained challenging behavior (e.g., differential reinforcement of compliance; Lipschultz & Wilder, 2017).

To illustrate how task changes may contribute to renewal, consider a treatment approach for task refusal involving different task types (e.g., Pálsdóttir et al., 2024). In this approach, treatment might begin with one type of academic task (e.g., reading) and after a period of low rates of challenging behavior and high rates of task completion, a new academic task, such as mathematics, might be introduced. The introduction of a new task (i.e., mathematics) may constitute a context change resulting in renewal of escape maintained challenging behavior.

Although task changes may represent a conceptual expansion of renewal in clinical work and influence prevalence estimates, it is also important to consider the methods used to measure renewal. In most applied studies, the same criterion has been used to determine whether renewal has occurred. Specifically, renewal is identified when the rate of challenging behavior in any of the three sessions immediately following a context change (i.e., *postchange* sessions) surpasses the high rate observed in the five sessions immediately preceding the change (i.e., *prechange* sessions; Muething et al., 2020). Recently, Podlesnik et al. (2025) suggested that this approach, which they termed the *max‐of‐5 criterion*, may be a conservative estimate of the prevalence of renewal that fails to detect renewal in some circumstances. They noted that early in treatment, challenging behavior may decrease from higher to lower rates, making the max‐of‐5 criterion less likely to capture renewal during or shortly after this transition. To address this issue, Podlesnik et al. retrospectively evaluated renewal data from two previously published studies (i.e., Falligant et al., 2021; Muething et al., 2020) using both the original max‐of‐5 criterion and a new *mean‐of‐2 criterion*, which compares the highest rate of the three postchange sessions with the mean of the two most proximal prechange sessions. Because the mean‐of‐2 criterion is less stringent, it identified approximately 8% more instances of renewal than the max‐of‐5 criterion. Given the difference in prevalence between the two criteria and the importance of considering different degrees of stringency, replication of these findings in additional data sets is warranted.

Overall, the prevalence of renewal following person and setting changes is well established in the literature. However, far less is known about renewal following task changes. In this study, we replicated previous research with person and setting changes, and we extended this work by also including an assessment of the prevalence and magnitude (i.e., level or strength of renewal) of task changes. Additionally, we calculated prevalence and magnitude using the max‐of‐5 and mean‐of‐2 criteria to evaluate the degree to which findings may differ when using criteria differing in stringency when defining renewal effects.

## METHOD

### Design and participants

This was a retrospective consecutive case series (Hagopian, 2020) study. We reviewed data from all consecutively encountered *cases*, defined as patient records, admitted to an intensive outpatient clinic for treatment of severe behavior between January 1, 2020, and December 31, 2023. *Participants* were defined as patients who met inclusion criteria: (a) they exhibited challenging behavior maintained solely by socially mediated variables based on a functional analysis (Iwata et al., 1982/1994), (b) they participated in a function‐based treatment evaluation (e.g., FCT) for three or more treatment visits and had at least 15 treatment sessions, (c) the treatment allowed for demonstration of experimental control, and (d) they experienced one or more context changes. One hundred and fifty‐eight cases were screened for inclusion. Twenty‐six cases were excluded from this study because their challenging behavior was exclusively or partially maintained by automatic reinforcement, 12 were excluded because they did not have three or more treatment visits or 15 or more treatment sessions, 12 were excluded because a functional analysis was not completed, and 10 were excluded because they did not have any context changes during treatment. This resulted in a sample of 98 participants (62.03% of all patients), ranging in age from 2 years 3 months to 27 years 0 months (*M* = 8.31 years, *SD* = 4.62 years). Table 2 summarizes demographic data across all participants who met inclusion criteria.

**TABLE 2:** Demographic characteristics of the study sample.

Characteristics	*N* = 98
Age in years, *M* ± SD	8.31 ± 4.62
Sex – *n* (%)	
Female	24 (24.49)
Male	74 (75.51)
Race – *n* (%)	
Asian	3 (3.06)
Black	8 (8.16)
White	81 (82.65)
Multiple races	6 (6.12)
Ethnicity – *n* (%)	
Hispanic or Latino	5 (5.10)
Not Hispanic or Latino	93 (94.90)
Cognitive level – *n* (%)	
Mild intellectual disability	30 (30.61)
Moderate intellectual disability	20 (20.41)
Severe/Profound intellectual disability	1 (1.02)
No intellectual disability	47 (47.96)
Diagnoses – *n* (%)	
Autism spectrum disorder	60 (61.22)
Attention deficit hyperactivity disorder	52 (53.06)
Communication disorder	53 (54.08)
Physical disability	17 (17.35)
Vocal communication skills – n(%)^a^	
Vocal	56 (57.14)
Minimally vocal	11 (11.22)
Nonvocal	31 (31.63)
Topographies of challenging behavior – *n* (%)	
Physical aggression	90 (91.84)
Destruction	86 (87.76)
Severe generalized refusal	64 (65.31)
Inappropriate vocalizations	58 (59.18)
Self‐injurious behavior	58 (59.18)
Elopement	47 (47.96)
Function of challenging behavior^b^	
Attention	28 (28.57)
Escape	70 (71.43)
Tangible	81 (82.65)
Multiple functions	68 (69.39)

*Note*: Participants could meet criteria for more than one function. “Multiple functions” indicates two or more identified functions.

^a^ Communication skills were categorized as follows: Vocal = 20 or more spoken words; minimally vocal = 5–20 spoken words; nonvocal = fewer than five spoken words.

^b^ Values reflect the number and percentage of participants with challenging behavior maintained by each function.

### General course of clinical care

Patients attended the intensive outpatient clinic in a university hospital for 3‐hr appointments, 5 days per week for 2–3 weeks (30–45 hr of assessment and treatment). Sessions took place in a large room at the university hospital. Clinicians included full‐time staff and graduate students who had completed rigorous training in behavioral assessment, treatment procedures, and safety protocols for managing challenging behavior. Clinicians were supervised by a licensed psychologist and board‐certified behavior analyst at the doctoral level. Safety measures were individualized to patients, including environmental modifications, protective equipment for staff or patients, and session termination criteria (e.g., self‐directed biting that broke the skin) when necessary.

For each patient, a functional analysis was conducted and resulted in the development of one or more function‐based treatments (e.g., FCT). Functions were determined based on visual inspection of the degree of differentiation between individual test conditions and the control condition. For patients with more than one unique function‐based treatment application, each treatment application was analyzed separately. Following demonstration of clinically meaningful reductions of challenging behavior with clinic staff and schedule thinning (when indicated), caregivers, school staff, or both were trained in treatment procedures using behavioral skills training.

Context changes were systematically introduced consistent with each patient's treatment plan (e.g., for generalization purposes). *Person changes* were recorded when any new treatment implementer (e.g., therapist, caregiver, teacher, paraprofessional) was introduced. Caregivers and school staff were often introduced during the later stages of treatment after observing treatment and learning the treatment procedures via behavioral skills training. *Setting changes* included the introduction of a new therapy room with different dimensions and physical arrangements of furniture, other locations on the hospital campus (e.g., waiting areas, library), a return to the home setting, and implementation in the school setting. Finally, *task changes* included any change in a primary academic subject (e.g., preacademic shape matching, mathematics, reading, writing) or domestic task (e.g., folding laundry, sorting silverware, picking up toys), and any change from an academic to domestic task or vice versa. Tasks were selected to be within each participant's existing skill repertoire and were not intended to systematically increase response effort or reduce programmed reinforcement density. Task changes were made upon reaching a terminal schedule for a particular task. Clinical judgment, with caregiver or teacher input, was then used to determine which tasks would be used for generalization. Although demand fading was often used, we did not include changes made to the task difficulty or quantity in our analysis, as this could have affected the comparability across participants (i.e., controlling for response effort). Thirty‐three concurrent context changes (e.g., a change in setting and person simultaneously) were excluded from analysis to ensure that effects could be attributed to a single type of context change.

Putative reinforcers were individualized for each patient based on preference assessments and aligned with the outcomes of the functional analysis. For example, a patient whose behavior was maintained by escape might receive an enriched break from work contingent on the alternative behavior, which could include access to a tablet and therapist attention if those were identified as preferred. Alternative responses targeted during treatment varied across patients but generally included functionally equivalent communication responses (e.g., requesting breaks, attention, or items) and compliance with tasks. The reinforcement schedules were held constant during all periods in which context changes occurred. Schedule thinning was completed prior to or after context changes rather than concurrently, including for patients with tangible maintained behavior. Thus, changes in schedule requirements did not overlap with person, setting, or task context changes.

### Data collection and analysis

Case files were screened for eligibility by three members of the research team using the previously described predefined inclusion and exclusion criteria. Prior to independent screening, screeners reviewed the criteria together and jointly evaluated a subset of cases to establish shared interpretation of rules. Each screener then independently reviewed assigned case files. Discrepancies in eligibility determinations were discussed during team meetings and resolved through consensus.

For the cases that met criteria, the same research team members extracted the following data: (a) demographics (e.g., age, biological sex, race, ethnicity, medical diagnoses); (b) behavioral topographies targeted for treatment; (c) identified function or functions; (d) rate of challenging behavior for each treatment session; and (e) all person, setting, and task context changes. Session data on challenging behavior and context changes during treatment were extracted from Microsoft Excel data files created when each patient was originally seen in the clinic. Challenging behavior data were collected using electronic data collection software (BDataPro; Bullock et al., 2017) and were recorded as responses per minute. All treatment sessions were 5 min.

### Outcome measures

#### Prevalence

We calculated prevalence as the number of context changes that resulted in renewal divided by the total number of context changes. This calculation was performed across all context changes and separately for person, setting, and task changes, using both the max‐of‐5 and mean‐of‐2 criteria. Using the max‐of‐5 criterion, renewal was identified when the rate of challenging behavior in any of the three postchange sessions exceeded the rates observed in each of the final five prechange sessions. When applying the mean‐of‐2 criterion, renewal was identified when the rates observed in any of the three postchange sessions exceeded the rates observed in each of the final two prechange sessions. For both criteria, the postchange session in which renewal was identified was coded as “Y” and all other sessions were coded as “N.”

#### Magnitude

Magnitude of renewal was calculated for the first five postchange sessions following a context change. We used a log‐transformation method initially introduced by Friedel et al. (2019) and more recently employed by Falligant et al. (2024) to calculate magnitude of renewal in automatically maintained challenging behavior. This method uses the log proportion rate of response to compare proportional changes from one session with those of another. Falligant et al. used this method to compare the dynamic session‐by‐session changes (e.g., final prechange vs. first postchange session, first postchange vs. second postchange session) as opposed to a simple proportion change of postchange sessions with a prechange value, which cannot be calculated with zero values. However, we modified the equation to compare each of the five postchange sessions to the mean response rate of the five prechange sessions. This was done to mirror the approach taken by Muething et al. (2020) and Falligant et al. (2021), who compared postchange sessions with the mean response rate of the five prechange sessions while also ensuring all data would be included. Thus, magnitude was calculated using the following formula:

(1) $$Y=\mathrm{lo}g_{2}\left(\frac{B_{n}+c}{{\bar{B}}_{\mathrm{pre}}+c}\right),$$

where *n* is a postchange session, $\bar{B}$_pre_ is the mean response rate across the five prechange sessions, *B*_*n*_ is the response rate for postchange session *n*, and *c* is a correction factor, which is set to 1. The correction factor prevents a denominator of zero when zero rates of challenging behavior are emitted during the prechange sessions, and it avoids taking the log of zero if *B*_*n*_ = 0. Like Falligant et al. (2024), the values from this equation show a proportional change in response rates, but in the present study this is proportional to the mean of the five prechange values. This method was applied to all occurrences of renewal, regardless of which renewal determination criterion was used.

#### Statistical analysis

Statistical analysis was conducted to characterize the rates at which renewal occurred across the different context change types (person, setting, and task). To do this we used two broad strategies. First, we conducted a descriptive analysis to report summary statistics for each of our objectives including prevalence and magnitude of renewal using both the max‐of‐5 and mean‐of‐2 criteria.

The second strategy involved statistical modeling to characterize the renewal rate as a function of context change type for both max‐of‐5 and mean‐of‐2 criteria. All statistical procedures were performed using the R statistical program (R Core Team, 2024). Prevalence was transformed into a binary variable in which “Y” sessions were coded as 1 and “N” sessions were coded as 0. Given the binary nature of the dependent variable, we fit a generalized linear mixed model with a binomial distribution and a logit link function. This approach was chosen to incorporate random effects that model variability between participants. Further, we conducted post hoc comparisons of renewal across the different contexts using the emmeans R package (Lenth, 2015). Modeling was performed using the generalized linear mixed‐effects regression method included in the lme4 package (Bates et al., 2015). For magnitude of renewal, a linear mixed model was fit to examine the effects of postchange session and context on renewal magnitude. We conducted post hoc comparisons of renewal magnitude across different contexts and across different postchange sessions.

### Interrater agreement

Two independent raters collected data on 100% of all variables (including both sets of renewal criteria) for context changes (presence and type), presence or absence of renewal, and rate of challenging behavior in each of the five prechange sessions and each of the five postchange sessions. For each variable, data were coded as agreement or disagreement. The number of agreements was summed and divided by the total number of agreements and disagreements. The quotient was then multiplied by 100 to obtain the percentage of agreement. Interrater agreement was 98.90% for all context changes (99.30% of person changes, 100% of setting changes, and 97.80% of task changes), 98.49% for presence or absence of renewal, and 96.89% for rate of challenging behavior across all prechange and postchange sessions. All disagreements were discussed between the first three authors and corrected such that final agreement was 100%.

## RESULTS

Across 98 participants and 139 unique function‐based treatment applications, there were 749 context changes, with every participant experiencing at least one (*M* = 7.64, *SD* = 5.33, range: 1–21). Among all 749 context changes, there were 405 person changes (54.07% of all context changes), 96 setting changes (12.82%), and 248 task changes (33.11%). Thirty‐five participants (35.71%) did not experience a task change (most often due to the absence of an escape function); however, for the 63 that did, there was an average of 3.94 task changes per participant.

### Prevalence

Table 3 provides prevalence data at the individual level. Using the max‐of‐5 criterion to calculate renewal, most participants (71.43%) demonstrated at least one renewal incident, with a similar proportion of participants experiencing renewal following person changes (56.99%) and task changes (57.14%). Less than half (38.89%) experienced renewal following setting changes. The total percentage of participants experiencing renewal increased to 84.69% when using the mean‐of‐2 criterion, with the highest percentage experiencing renewal following person changes (76.34%).

**TABLE 3:** Comparison of renewal prevalence between max‐of‐5 and mean‐of‐2 criteria: Participant‐level outcomes.

Context change	Patients with ≥1 context changes	Patients demonstrating renewal *n* (%)	
		Max‐of‐5 criterion	Mean‐of‐2 criterion
Overall	98	70 (71.43)	83 (84.69)
Person	93	53 (56.99)	71 (76.34)
Setting	36	14 (38.89)	19 (52.78)
Task	63	36 (57.14)	43 (68.25)

Table 4 shows the overall prevalence of renewal and the prevalence across each type of context change using the max‐of‐5 and mean‐of‐2 criteria. Based on the max‐of‐5 criterion, overall prevalence was 25.23%. The highest prevalence of renewal, at 27.02%, occurred following task changes. Slightly lower prevalence was observed following changes to person (24.69%) and setting (22.92%).

**TABLE 4:** Comparison of renewal prevalence between max‐of‐5 and mean‐of‐2 criteria: Context‐change‐level outcomes.

Context change	Total number of context changes	Prevalence		Absolute increase from max‐of‐5 to mean‐of‐2	Percentage of increase from max‐of‐5 to mean‐of‐2
		Max‐of‐5 criterion *n* (%)	Mean‐of‐2 criterion *n* (%)		
Overall	749	189 (25.23)	298 (39.79)	14.56	57.71
Person	405	100 (24.69)	160 (39.51)	14.82	60.02
Setting	96	22 (22.92)	35 (36.46)	13.54	59.08
Task	248	67 (27.02)	103 (41.53)	14.51	53.70

Application of the mean‐of‐2 criterion resulted in a higher prevalence of renewal overall and across each context change when compared with the max‐of‐5 criterion, likely because the max‐of‐5 criterion produces higher thresholds for renewal than the mean‐of‐2 criterion. The overall mean‐of‐2 prevalence of renewal was 39.79%, which is a 57.71% increase relative to the max‐of‐5 criterion. Like the max‐of‐5 criterion, the highest prevalence of renewal was for task changes (41.53%) and was followed by changes to person (39.51%) and setting (36.46%). The prevalence for each type of context change was at least 50% higher than that obtained using the max‐of‐5 criterion, with the greatest increase (60.02% relative increase) associated with person changes.

Supporting Information A includes the results of the mixed‐effects analysis for the max‐of‐5 and mean‐of‐2 criteria. For the max‐of‐5 criterion, neither task (β = 0.17, SE = 0.31, *z* = 0.54, *p* = .588) nor person (β = 0.05, SE = 0.30, *z* = 0.19, *p* = .852) context changes showed a significant difference in prevalence of renewal relative to setting. That is, no differences in the prevalence of renewal were observed for setting context changes relative to person or task context changes. We performed pairwise post hoc tests to further evaluate differences between context change types. Supporting Information B presents results for the max‐of‐5 and mean‐of‐2 criteria. No significant differences were observed between the different context change types (*z*s ≤ 0.57, *p*s ≥ .838). Last, the results suggested no meaningful differences were found between task and setting changes versus person changes (OR ≤ 1.12).

For the mean‐of‐2 criterion, neither task (β = 0.26, SE = 0.29, *z* = 0.92, *p* = .358) nor person (β = 0.15, SE = 0.27, *z* = 0.56, *p* = .578) context changes showed a significant difference in prevalence of renewal relative to setting (Supporting Information A). That is, no differences in the prevalence of renewal were observed for setting context changes relative to person or task context changes. Post hoc tests of pairwise comparisons between context change types suggested no significant differences were observed between the different context change types (Supporting Information B; *z* ≤ 0.622, *p* ≥ 0.628). Last, the results suggested no meaningful differences between context changes (OR ≤ 1.12).

### Magnitude

**FIGURE 1:**
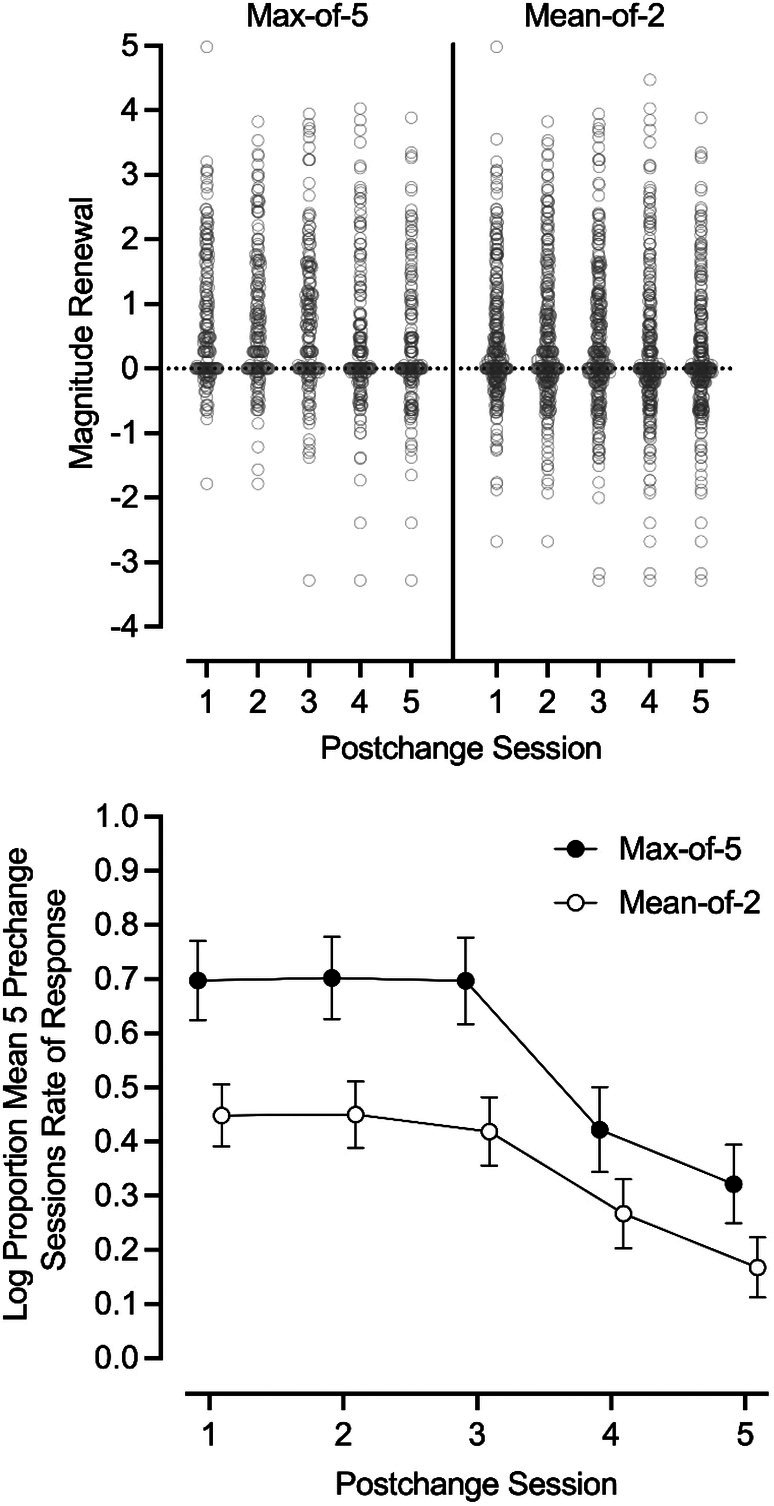
Magnitude of renewal effects across postchange sessions based on max‐of‐5 and mean‐of‐2 criteria. Top panel: Bee‐swarm plot of renewal magnitude by postchange session. Each data point represents individual participant data. Bottom panel: Each data point represents the mean log proportion response rate across all context change types resulting in renewal. Errors bars represent standard error of the mean.

The top panel of Figure 1 displays individual magnitude data for all participants across postchange sessions for both criteria. The bottom panel of Figure 1 displays the mean magnitude of renewal across each of the five postchange sessions for all instances of renewal. Relative to the mean prechange value, the magnitude of renewal was consistently higher across all postchange sessions under the max‐of‐5 criterion. This difference is solely attributable to the added renewal events identified using the mean‐of‐2 criterion (109 additional instances of renewal). Thus, lower magnitudes using the mean‐of‐2 criterion reflect the lower magnitude of these additional renewal events that were not identified as instances of renewal with the max‐of‐5 criterion.

For both renewal criteria, renewal magnitude was comparable during the first three postchange sessions and then declined steadily across the final two sessions. Despite this decline, challenging behavior did not return to the prechange mean by the fifth postchange session for either criterion.

**FIGURE 2:**
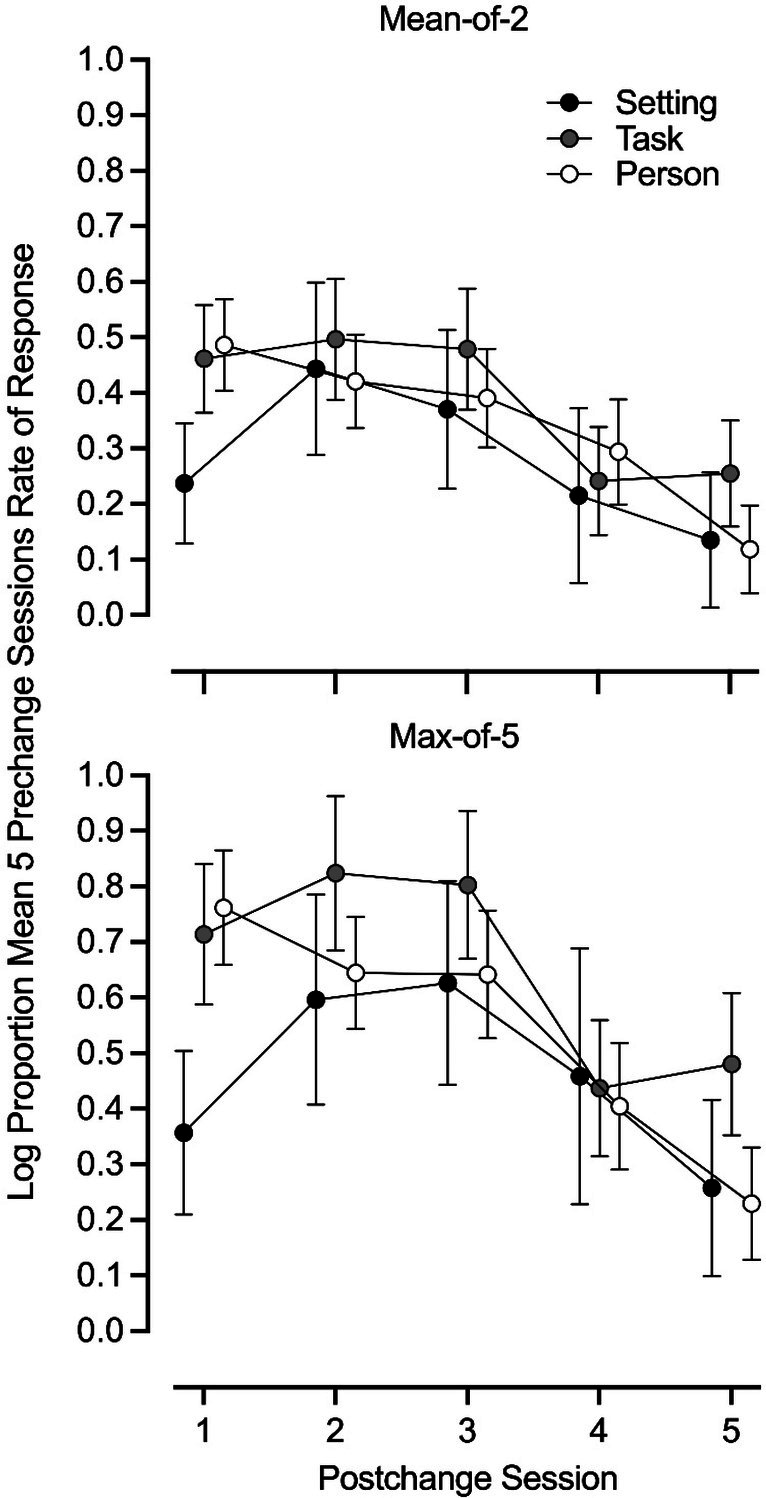
Magnitude of renewal effects across postchange sessions by type of context change. Each data point represents the mean log proportion response rate across one context change type resulting in renewal. Errors bars represent standard error of the mean.

Figure 2 shows the mean magnitude of renewal across the five postchange sessions for each type of context change using the mean‐of‐2 criterion (upper panel) and the max‐of‐5 criterion (lower panel). The magnitude of renewal following task changes was generally higher than what was observed for setting and person changes. Similar to the overall magnitude data, rates of challenging behavior did not return to prechange levels by the fifth postchange session for any of the three context changes.

Supporting Information C includes the results of the linear mixed model for the max‐of‐5 and mean‐of‐2 criteria. Supporting Information D includes plots of linear mixed‐effects model residuals. Visual inspection of model residuals indicated some deviation from normality and homoscedasticity, suggesting that model assumptions may not be fully met. Therefore, results should be interpreted with caution.

For both criteria, the magnitude of renewal decreased significantly from the first postchange session to the fourth and fifth postchange sessions (Lines 3–4 and 11–12 in Supporting Information C). Supporting Information E shows results of post hoc tests of pairwise comparisons between postchange sessions regardless of type of context for both criteria. The results emphasized the model results and showed significant differences were observed between the first postchange session and the fourth and fifth postchange sessions (Lines 3–4 and 13–14). In other words, the magnitude of the first postchange session was significantly greater than the magnitude of the fourth and fifth postchange sessions for both the mean‐of‐2 and max‐of‐5 criterion. Additionally, there was a statistically significant difference between the second postchange session and the fourth and fifth postchange sessions (Lines 6–7 and 16–17). That is, the magnitude of the second postchange session was significantly greater than the magnitude of the fourth and fifth postchange sessions. For the mean‐of‐2 criterion, the magnitude of renewal was statistically significantly greater in the third postchange session than in the fifth postchange session (Line 9). Moreover, for the max‐of‐5 criterion, the magnitude of renewal was statistically significantly greater in the third postchange session than in the fourth and fifth postchange sessions (Lines 18–19). No other significant postchange session pairwise comparisons were observed. Last, Supporting Information F includes outcomes from the pairwise comparisons of magnitude for all criteria. Results revealed no significant differences between context types regardless of postchange session.

## DISCUSSION

**FIGURE 3:**
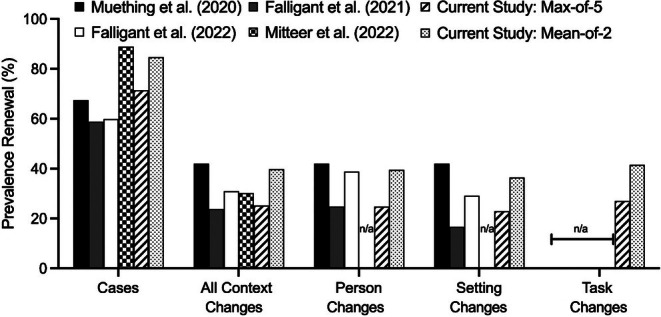
Prevalence of renewal by case and context change across studies. N/A = not applicable, indicating that the variable was not assessed or reported in the study.

In this study of 98 participants, we replicated previous findings showing that renewal commonly follows person and setting changes (i.e., Falligant et al., 2021, 2022; Muething et al., 2020) and extended this work by evaluating renewal following task changes. Renewal prevalence using the max‐of‐5 criterion was 25.23% (39.79% using the mean‐of‐2 criterion), which is lower than the prevalence observed by Muething et al. (2020; 42.31%) but in line with studies by Falligant et al. (2021; 23.50%), Falligant et al. (2022; 30.54%), and Mitteer et al. (2022; 30.19%), all of whom used similar methodologies and determination criteria. As shown in Figure 3, renewal following setting and person changes was within the range of outcomes reported by previous studies.

Task changes accounted for one third of all context changes. The prevalence of renewal following task changes exceeded the prevalence of renewal following either setting or person changes, although the differences were not statistically significant. Additionally, the magnitude of renewal following task changes was higher than the magnitude for person or setting changes when using the max‐of‐5 criterion and similar to that of person changes when using the mean‐of‐2 criterion. These outcomes underscore the importance of including task changes in renewal research and should raise clinicians' awareness of the possibility of renewal following task changes in behavioral treatments.

There is a possibility that some task changes, particularly those that are perceived as more difficult, effectively reduce the rate of reinforcement even when reinforcement contingencies remain unchanged. As task difficulty increases, learners may respond more slowly, emit more errors, or fail to meet reinforcement criteria at the same rate as with prior tasks, thereby experiencing a functional thinning of reinforcement. This would represent a worsening of reinforcement conditions and could result in a resurgence of challenging behavior (see Mitteer et al., 2018; Podlesnik et al., 2017; Williams et al., 2023). The combination of context changes and worsening of reinforcement conditions may have contributed to the current finding that prevalence was greater with task changes than with person or setting context changes.

The present findings underscore the importance of considering skill acquisition and skill deficits in relation to behavioral lapse. Individuals with limited repertoires of alternative skills may be particularly vulnerable to behavioral lapse when treatment conditions shift. Prior work on FCT has shown that durability of treatment effects depends in part on whether alternative responses are robust, generalized, and resistant to disruption (Hagopian et al., 2011; Nevin & Wacker, 2013; Tiger et al., 2008; Wacker et al., 2017). Thus, the availability and strength of alternative skills likely interact with task changes to influence the likelihood of a lapse.

It is worth noting that although task changes are common in behavioral treatments, there is potential for many other context changes to influence challenging behavior. As Bouton (2019) has suggested, context may be much more than what is easily perceived by a clinician or researcher. This might be related to *stimulus overselectivity*, a phenomenon in which only some of the stimuli in the environment evoke responding. Stimulus overselectivity has been demonstrated among individuals with autism (e.g., Kelly & Reed, 2021) and impedes learning, as the individual may attend to only a subset of the stimuli in the environment. The individual who only focuses on parts of the learning environment may have a higher likelihood of challenging behavior when there are idiosyncratic and nonidiosyncratic context changes. We chose to focus on context changes that were relatively easy to observe and document (i.e., task, person, setting), but there likely were many other changes we did not or could not observe (e.g., change in room temperature, change in noise in an adjacent room, food deprivation or drug states). As researchers and clinicians, we may need to broaden our awareness of contextual variables that could contribute to renewal beyond treatment implementers, settings, and tasks.

Our findings regarding renewal magnitude revealed a nuanced pattern following context changes. Previous research has yielded mixed results in this area, with some studies showing a steady decline in the magnitude across successive postchange sessions (e.g., Mitteer et al., 2022; Muething et al., 2020) and others not (e.g., Falligant et al., 2021). The lack of consistent findings may be due to variations in how the magnitude of renewal has been calculated across studies. In the current analysis, we calculated renewal magnitude using a log‐proportion transformation, similar to Falligant et al. (2024), but we used the mean rate of challenging behavior in prechange sessions for comparison. This approach improved sensitivity to within‐subject changes and accounted for situations in which the prechange rate of challenging behavior was zero. Using this method, we found no appreciable difference in renewal magnitude between the first and second postchange sessions but a consistent decrease in magnitude across the third through fifth sessions. These findings partially align with prior reports of declining renewal but also suggest that reductions in magnitude may be delayed rather than immediate. Notably, the magnitude data show the mean rates of challenging behavior following context changes failed to reach the prechange levels by the fifth postchange session. This underscores the importance of continued procedural fidelity and calls for further research on the persistence of renewal effects. As a start, a reanalysis of all published renewal data with the different calculations of magnitude to identify or rule out the influence of calculation method on persistence is warranted.

The main results from this study concerning the two renewal determination criteria varied little. The overall and context‐specific prevalence of renewal was lower when using the max‐of‐5 criterion, and the magnitude of renewal was higher when using the mean‐of‐2 criterion. However, the results from the max‐of‐5 criterion paralleled those from the mean‐of‐2 criterion in several ways. For example, the relative difference between highest and lowest renewal prevalence across context change types was comparable for the two criteria (17.89% for max‐of‐5 criterion and 13.91% for mean‐of‐2 criterion), as was the pattern of magnitude change across the five postchange sessions. Additionally, according to each criterion, the lowest prevalence of renewal was associated with setting changes. Thus, although the mean‐of‐2 criterion provided a less stringent criterion for determining the prevalence of renewal, it did not illuminate any distinct patterns not observed with the max‐of‐5 criterion. Consistent with the present findings, Podlesnik et al. (2025) reported higher estimates of renewal when using the mean‐of‐2 criterion than when using the more conservative max‐of‐5 criterion, highlighting how renewal prevalence is sensitive to the operational definition used.

As a preliminary investigation of renewal following task changes, this study is not without limitations. First, tasks were not empirically evaluated for level of difficulty (e.g., a demand assessment) or preference across patients. The clinical teams chose tasks within each patient's skill set based on interviews with caregivers and teachers as well as observation during the assessment. Thus, it is possible that some instances of renewal that followed a task change were a result of a perceived change in task difficulty or task preference rather than task type. Conceptually, such effects might resemble resurgence, whereas task substitutions that alter contextual features are more consistent with renewal (see King et al., 2024, for a relevant discussion). Future studies should examine whether equivalence (in difficulty and preference) can be established between different types of tasks to determine whether increases in challenging behavior are related to task changes as contextual variables or changes in perceived difficulty or preference as contingency variables.

Second, as with other retrospective analyses of renewal, we summarized all instances of renewal and did not account for potential differences in renewal as a result of transitioning to original versus novel contexts. Basic research studies (e.g., Bouton et al., 2011) suggest stronger renewal effects when treatment returns to the original context in which the behavior was reinforced (ABA renewal) than a novel context (ABC renewal; but see Todd, 2013). The prevalence of renewal across retrospective analyses might be skewed by differences in the unreported types of context changes arranged during treatment. Future research should consider categorizing the types of context changes arranged during treatment as transitions to original or novel contexts to further analyze the influence that context type has on measures of renewal.

Third, we did not evaluate the fidelity of treatment implementation by clinic therapists or novel treatment implementers. Therapists in this clinic are highly trained, whereas novel implementers (e.g., caregivers) are trained to mastery criteria prior to implementing treatment independently. All treatment implementers (including caregivers, school staff, and novel therapists) received behavioral skills training and in vivo coaching and feedback via a “bug‐in‐the‐ear” device. Nonetheless, fidelity errors are still possible. In fact, increases in challenging behavior resulting from a context change might increase the likelihood of fidelity breakdowns, which in turn may lead to other forms of lapse or relapse (see Mitteer et al., 2018; Williams et al., 2023). Absent of a context change, an implementer may still inadvertently fail to provide a scheduled reinforcer following an alternative response (e.g., a mand during FCT), mistakenly provide a reinforcer noncontingently, or provide a reinforcer contingent on the occurrence of challenging behavior, each of which contribute to other lapses in behavioral treatments (resurgence, reinstatement, and reacquisition, respectively; see Wathen & Podlesnik, 2018, for a review). Future research should evaluate whether renewal might be confounded with other fidelity errors and whether renewal induces other forms of treatment lapse to further isolate the effects of renewal (Podlesnik et al., 2017) versus threats to procedural fidelity (St. Peter, 2023).

Unlike some forms of lapse in behavioral treatments, renewal represents a “no fault” form of treatment lapse: Even when procedural fidelity is high, context changes may result in increased rates of challenging behavior. Nevertheless, our data and the findings from others (Mitteer et al., 2022; Muething et al., 2020) show that with continued treatment implementation in the face of renewal, the initial increase in challenging behavior is followed by steady reduction across successive sessions. Thus, when treatment can be provided with high fidelity, behavior increases due to context changes may resemble a brief “lapse” rather than a prolonged “relapse” (see Brown, 2025, for discussion). Accordingly, in addition to strategies to mitigate the occurrence of renewal, research should focus on strategies that reduce the magnitude and persistence of renewal. In doing so, renewal encountered by nonclinicians (e.g., caregivers, teachers, group home staff) would minimize threats to procedural fidelity and ensure challenging behavior returns to prechange levels. Several researchers have offered insights, mostly grounded in basic research, on factors associated with larger renewal effects, including higher baseline response rate, greater disparity between treatment and renewal contexts, and simultaneous context changes (Kimball et al., 2023; Podlesnik et al., 2017). Future research should consider how these factors might be leveraged to minimize renewal magnitude and persistence.

Further research on mitigation strategies seems crucial for minimizing renewal magnitude and persistence, thereby enhancing the long‐term maintenance of treatment effects. In fact, several reviews and studies have proposed or evaluated several mitigation strategies (e.g., Kelley et al., 2018; Kimball et al., 2023; Podlesnik et al., 2017; Silva & Saini, 2025; Wathen & Podlesnik, 2018), including programming for generalization, fading in new context stimuli, and including stimuli indicating extinction is present. Although many of these strategies are commonly used in clinical practice, including the clinic in the present study, research on renewal mitigation has primarily come from laboratory studies with nonhuman animals and typically in the absence of treatments based on differential reinforcement (e.g., Bernal‐Gamboa et al., 2017), with only a few notable exceptions from the applied literature (e.g., Bernal‐Gamboa et al., 2020; Greer et al., 2019; see examples in Kimball & Kranak, 2022). Accordingly, additional applied research is needed to evaluate the feasibility and effectiveness of these strategies in clinical practice.

### AUTHOR CONTRIBUTIONS

The first author (AI) contributed to conceptualization, led data curation, assisted with methodology development, and contributed to manuscript drafting and revisions. The second author (MJO) conceptualized the study, led development of the methodology, supervised and administered the project, contributed to data curation, drafted the initial manuscript, and led manuscript revisions. The third author (AMP) assisted with data curation, formal analysis, and methodology development and contributed to manuscript drafting and revisions. The fourth author (SRJ) contributed to methodology development and assisted with manuscript drafting and revisions. The fifth author (CTF) led the formal analysis and contributed to methodology development, manuscript drafting, and revisions. The sixth author (CNMP) contributed to conceptualization, formal analysis, and methodology development; led visualization; and assisted with manuscript drafting and revisions. The seventh author (CAP) contributed to conceptualization, methodology development, supervision, and manuscript drafting and revisions. All authors reviewed, discussed, and approved the final manuscript and agreed to be accountable for all aspects of the work.

## CONFLICT OF INTEREST STATEMENT

There is no conflict of interest to declare.

## ETHICS APPROVAL

This study received institutional review board approval and was conducted in accordance with established ethical guidelines for the treatment of human participants. Specifically, this study was approved by the Human Subjects Office/IRB at the University of Iowa.

## Supporting information


**Supporting Information A** *Results of Mixed‐Effects Modeling of Prevalence for All Criteria*.
**Supporting Information B**. *Context Pairwise Comparisons of Prevalence for All Criteria*.
**Supporting Information C**. *Results of Mixed‐Effects Modeling of Magnitude for All Criteria*.
**Supporting Information D**. *Plots of Linear Mixed‐Effects Models Residuals for All Criteria*.
**Supporting Information E**. *Post‐Change Session Pairwise Comparisons of Magnitude for All Criteria*.
**Supporting Information F**. *Context Pairwise Comparisons of Magnitude for All Criteria*.

## Data Availability

The data that support the findings of this study are available from the corresponding author upon reasonable request. Supporting Information includes supplemental tables, figures, statistical model outputs, post hoc comparisons, and diagnostic plots referenced in the Results section. Specifically, Supporting Information A–F provide mixed‐effects model results for renewal prevalence and magnitude, pairwise comparison results, residual diagnostics for linear mixed‐effects models, and additional analyses supporting interpretation of renewal patterns across context change types.

## Footnotes

^1^
We use the term “lapse” rather than “relapse” when referring to renewal, following the argument made by Brown (2025) that a lapse reflects a transient increase in challenging behavior, whereas relapse denotes a more prolonged increase.
