# Supplementary material for: Renewal of challenging behavior in an intensive outpatient clinic: Replication and extension to task changes
Source: J Appl Behav Anal. 2026 Feb 11;59(2):e70057. doi: 10.1002/jaba.70057 (PMC12895135; doi:10.1002/jaba.70057)
Supplement: Supplementary file 1 — Supporting Information A Results of Mixed‐Effects Modeling of Prevalence for All Criteria. Supporting Information B. Context Pairwise Comparisons of Prevalence for All Criteria. Supporting Information C. Results of Mixed‐Effects Modeling of Magnitude for All Criteria. Supporting Information D. Plots of Linear Mixed‐Effects Models Residuals for All Criteria. Supporting Information E. Post‐Change Session Pairwise Comparisons of Magnitude for All Criteria. Supporting Information F. Context Pairwise Comparisons of Magnitude for All Criteria. [file JABA-59-0-s001.docx]

**Supporting Information**

**Supporting Information A**

*Results of Mixed-Effects Modeling of Prevalence for All Criteria*

| **Fixed Effect** | **𝛽^** | **SE** | ***z*** | ***p* value** |
| --- | --- | --- | --- | --- |
| **Max-of-5 Criterion** |  |  |  |  |
| (Intercept) | -1.26 | 0.28 | -4.53 | <.001 |
| Task | 0.17 | 0.31 | 0.54 | .588 |
| Person | 0.05 | 0.30 | 0.19 | .852 |
| **Mean-of-2 Criterion** |  |  |  |  |
| (Intercept) | -0.62 | 0.25 | -2.45 | .014 |
| Task | 0.26 | 0.29 | 0.92 | .358 |
| Person | 0.15 | 0.27 | 0.56 | .578 |

*Note.* SE = standard error of the mean.

**Supporting Information B**

*Context Pairwise Comparisons of Prevalence for All Criteria*

| **Contrast** | **Odds ratio** | **SE** | ***z*** | ***p* value** |
| --- | --- | --- | --- | --- |
| **Max-of-5 Criterion** |  |  |  |  |
| Setting vs. Task | 0.84 | 0.27 | -0.54 | .852 |
| Setting vs. Person | 0.95 | 0.28 | -0.19 | .981 |
| Task vs. Person | 1.12 | 0.23 | 0.57 | .838 |
| **Mean-of-2 Criterion** |  |  |  |  |
| Setting vs. Task | 0.77 | 0.22 | -0.92 | .628 |
| Setting vs. Person | 0.86 | 0.23 | -0.56 | .843 |
| Task vs. Person | 1.12 | 0.21 | 0.62 | .810 |

*Note.* SE = standard error of the mean.

**Supporting Information C**

*Results of Mixed-Effects Modeling of Magnitude for All Criteria*

| **Line** | **Fixed Effects** | **𝛽^** | **SE** | **df** | ***t*** | ***p* value** |
| --- | --- | --- | --- | --- | --- | --- |
|  | **Mean-of-2 Criterion** |  |  |  |  |  |
| 1 | (Intercept) | 0.459 | 0.112 | 552.5 | 4.07 | <.001 |
| 2 | Post-Change Session 2 | 0.001 | 0.079 | 1396 | 0.01 | .987 |
| 3 | Post-Change Session 3 | -0.029 | 0.079 | 1396 | -0.37 | .708 |
| 4 | Post-Change Session 4 | -0.181 | 0.079 | 1396 | -2.30 | .022 |
| 5 | Post-Change Session 5 | -0.281 | 0.079 | 1396 | -3.55 | <.001 |
| 6 | Task | -0.036 | 0.105 | 1085 | -0.34 | .731 |
| 7 | Person | -0.063 | 0.100 | 1091 | -0.63 | .524 |
|  | **Max-of-5 Criterion** |  |  |  |  |  |
| 8 | (Intercept) | 0.697 | 1.024 | 788.7 | 0.68 | .496 |
| 9 | Post-Change Session 2 | 0.005 | 0.098 | 886.7 | 0.04 | .962 |
| 10 | Post-Change Session 3 | <-0.001 | 0.098 | 886.7 | -0.01 | .995 |
| 11 | Post-Change Session 4 | -0.275 | 0.098 | 886.7 | -2.78 | .005 |
| 12 | Post-Change Session 5 | -0.375 | 0.098 | 886.7 | -3.80 | < .001 |
| 13 | Task | -0.054 | 1.033 | 782.2 | -0.05 | .958 |
| 14 | Person | -0.020 | 1.029 | 784.7 | -0.02 | .984 |

*Note.* SE = standard error of the mean; df = degrees of freedom.

**Supporting Information D**

*Plots of Linear Mixed-Effects Models Residuals for All Criteria*


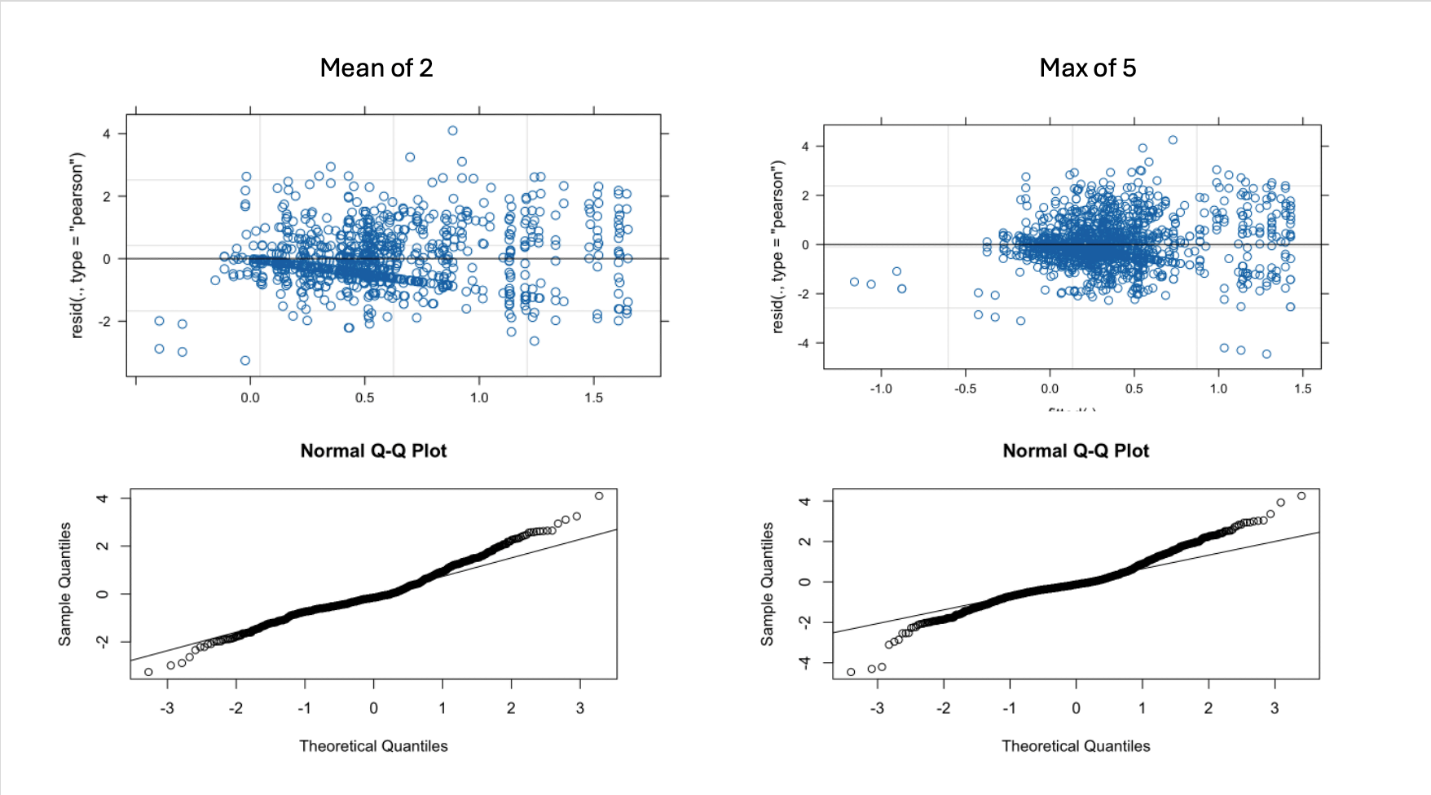


**Supporting Information E**

*Post-Change Session Pairwise Comparisons of Magnitude for All Criteria*

| **Line** | **Contrast** |  |  | **Estimate** | **SE** | **df** | **t** | ***p* value** | |  |
| --- | --- | --- | --- | --- | --- | --- | --- | --- | --- | --- |
|  | **Mean-of-2 Criterion** |  |  |  |  |  |  |  |  |  |
| 1 | Post-Change Session 1 | - | Post-Change Session 2 | -0.001 | 0.08 | 1405 | -0.02 | >.999 |  |  |
| 2 | Post-Change Session 1 | - | Post-Change Session 3 | 0.03 | 0.08 | 1405 | 0.38 | .996 |  |  |
| 3 | Post-Change Session 1 | - | Post-Change Session 4 | 0.18 | 0.08 | 1405 | 2.30 | .145 |  |  |
| 4 | Post-Change Session 1 | - | Post-Change Session 5 | 0.28 | 0.08 | 1405 | 3.55 | .004 |  |  |
| 5 | Post-Change Session 2 | - | Post-Change Session 3 | 0.03 | 0.08 | 1405 | 0.39 | .995 |  |  |
| 6 | Post-Change Session 2 | - | Post-Change Session 4 | 0.18 | 0.08 | 1405 | 2.32 | .140 |  |  |
| 7 | Post-Change Session 2 | - | Post-Change Session 5 | 0.28 | 0.08 | 1405 | 3.57 | .003 |  |  |
| 8 | Post-Change Session 3 | - | Post-Change Session 4 | 0.15 | 0.08 | 1405 | 1.93 | .305 | | |
| 9 | Post-Change Session 3 | - | Post-Change Session 5 | 0.25 | 0.08 | 1405 | 3.18 | .013 | | |
| 10 | Post-Change Session 4 | - | Post-Change Session 5 | 0.09 | 0.08 | 1405 | 1.25 | .719 | |  |
|  | **Max-of-5 Criterion** |  |  |  |  |  |  |  |  |  |
| 11 | Post-Change Session 1 | - | Post-Change Session 2 | -0.0005 | 0.09 | 873 | -0.04 | >.999 |  |  |
| 12 | Post-Change Session 1 | - | Post-Change Session 3 | 0.0006 | 0.09 | 873 | 0.01 | >.999 |  |  |
| 13 | Post-Change Session 1 | - | Post-Change Session 4 | 0.27 | 0.09 | 873 | 2.79 | .043 |  |  |
| 14 | Post-Change Session 1 | - | Post-Change Session 5 | 0.36 | 0.09 | 873 | 3.80 | .001 |  |  |
| 15 | Post-Change Session 2 | - | Post-Change Session 3 | 0.005 | 0.09 | 873 | 0.05 | >.999 |  |  |
| 16 | Post-Change Session 2 | - | Post-Change Session 4 | 0.28 | 0.09 | 873 | 2.83 | .038 |  |  |
| 17 | Post-Change Session 2 | - | Post-Change Session 5 | 0.38 | 0.09 | 873 | 3.85 | .001 |  |  |
| 18 | Post-Change Session 3 | - | Post-Change Session 4 | 0.27 | 0.09 | 873 | 2.78 | .044 |  |  |
| 19 | Post-Change Session 3 | - | Post-Change Session 5 | 0.37 | 0.09 | 873 | 3.79 | .002 |  |  |
| 20 | Post-Change Session 4 | - | Post-Change Session 5 | 0.10 | 0.09 | 873 | 1.02 | .847 |  |  |

*Note.* SE = standard error of the mean; df = degrees of freedom.

**Supporting Information F**

*Context Pairwise Comparisons of Magnitude for All Criteria*

| **Contrast** | **Estimate** | **SE** | ***df*** | **t** | ***p* value** |
| --- | --- | --- | --- | --- | --- |
| **Mean-of-2 Criterion** |  |  |  |  |  |
| Setting vs. Task | 0.04 | 0.11 | 1117 | 0.34 | .937 |
| Setting vs. Person | 0.06 | 0.10 | 1123 | 0.63 | .801 |
| Task vs. Person | 0.03 | 0.07 | 1145 | 0.40 | .913 |
| **Max-of-5 Criterion** |  |  |  |  |  |
| Setting vs. Task | -0.03 | 0.14 | 472 | -0.25 | .967 |
| Setting vs. Person | 0.09 | 0.13 | 514 | 0.74 | .742 |
| Task vs. Person | 0.13 | 0.08 | 549 | 1.51 | .286 |

*Note.* SE = standard error of the mean; df = degrees of freedom.
